# Patterns of wild carnivore attacks on humans in urban areas

**DOI:** 10.1038/s41598-018-36034-7

**Published:** 2018-12-07

**Authors:** Giulia Bombieri, María del Mar Delgado, Luca Francesco Russo, Pedro José Garrote, José Vicente López-Bao, José M. Fedriani, Vincenzo Penteriani

**Affiliations:** 1 Research Unit of Biodiversity (UMIB, UO-CSIC-PA), Oviedo University - Campus Mieres, Mieres, Spain; 2 Museo delle Scienze, Sezione Zoologia dei Vertebrati, Corso del Lavoro e della Scienza 3, I-38123 Trento, Italy; 3 Centre for Applied Ecology “Prof. Baeta Neves”/InBIO, Institute of Agronomy, University of Lisbon, Tapada da Ajuda, Lisboa Portugal; 4 Instituto Pirenaico de Ecología, C.S.I.C., Avda. Nuestra Señora de la Victoria 16, 22700 Jaca, Spain

## Abstract

Attacks by wild carnivores on humans represent an increasing problem in urban areas across North America and their frequency is expected to rise following urban expansion towards carnivore habitats. Here, we analyzed records of carnivore attacks on humans in urban areas of the U.S. and Canada between 1980 and 2016 to analyze the general patterns of the attacks, as well as describe the landscape structure and, for those attacks occurring at night, the light conditions at the site of the attacks. We found that several behavioral and landscape-related factors were recurrent elements in the attacks recorded. The species for which the attack locations were available (coyote and black bear) attacked in areas with different conditions of landscape structure and artificial light. Specifically, black bears attacked more frequently in areas with abundant and aggregated vegetation cover and scarce buildings and roads, while coyotes attacked in a broader range of landscape conditions. At night, black bears attacked in generally darker areas than coyotes. By providing a comprehensive perspective of the phenomenon, this study will improve our understanding of how effective strategies aimed at reducing the frequency of risky encounters in urban areas should be developed.

## Introduction

Recent years have witnessed an increase in conflicts between humans and wild carnivores in North American urban areas (i.e., populated places, defined by the U.S. Geological Survey (https://www.usgs.gov/) as “*a place or area with clustered or scattered buildings and a permanent human population (city*, *settlement*, *town*, *village)*”)^1,2^. These conflicts include property damage, anthropogenic food consumption, livestock and pet attacks and, more rarely, attacks on people^1,3–5^. Increasing overlap between human and carnivore habitats may be behind this trend^6,7^. On the one hand, some populations of carnivores have expanded their range due to the improved human attitudes and stricter protection in recent years. On the other hand, the rapid expansion of urban areas into landscapes inhabited by these species is causing large areas of natural patches to surround or be incorporated into urban areas^6,8,9^. These natural patches provide carnivores with suitable habitats (e.g., abundance of prey and shelter) in close proximity to, or even inside, human developments. This, together with the ability of some carnivores to use anthropogenic resources (e.g., non-seasonal and high-caloric anthropogenic food) and thrive in highly human-modified landscapes may lead to increased conflictual interactions^10–12^.

Even though attacks on humans in urban areas are rare and mainly result in minor injuries, they often elicit lethal responses towards the animals considered responsible for the attack and decrease public tolerance towards these species, subsequently influencing management and conservation actions^6,13,14^. Therefore, both humans and carnivores lose when such incidents happen and, because of this, reducing the occurrence of such attacks in urban areas should be considered a priority for authorities. For this reason, rigorous analysis of attack scenarios aimed at identifying the factors which may drive risky human-carnivore encounters can provide decision-makers with useful information^13,14^.

Only a handful of studies have focused on wild carnivore attacks on humans in urban areas^1,15,16^. These studies have analyzed coyote *Canis latrans* attacks only and have highlighted that changes in human behaviors (e.g., management of attractants and pet supervision) can play a crucial role in reducing the number of attacks. However, several other factors need to be taken into consideration when analyzing attack triggers and scenarios. For example, information regarding the characteristics of the natural and human environment at the site of the attack, as well as the conditions of artificial illumination for those incidents that occurred at night, might turn out to be crucial for understanding the dynamics of the attacks and for the development of management actions aimed at reducing the risk of dangerous encounters. Moreover, although until recent years coyotes have been almost the only species responsible for attacks on humans in urban areas, the current increase in the number of attacks by other wild carnivores^17^ highlights the need for a more comprehensive approach encompassing all carnivore species occurring in urban landscapes.

Here, we analyzed the scenarios of carnivore attacks on humans that occurred in urban areas across the U.S. and Canada during the last 36 years (from 1980 to 2016). We first studied temporal patterns of the attacks at different scales (i.e., circadian, seasonal and annual) and general patterns related to various factors such as age and sex of the victims, party composition, location and scenario of the attacks. Further, we examined the structure of the landscape (i.e., abundance and structure of vegetation, abundance of buildings and roads) at the attack sites and assessed whether differences in attack patterns between species exist. Specifically, following what found in previous studies on other kinds of conflicts^4,5,18^, we hypothesized that species which are mostly forest-obligate and generally avoid humans will mainly attack under landscape conditions characterized by high vegetation cover and the fewest human infrastructures, whereas we expected landscape structure to not be relevant for those species which are known to reside in urban environments and tolerate human presence. Finally, for those attacks occurring at night, we explored whether (and how) light conditions might influence the occurrence of an attack. Specifically, we hypothesized that a higher number of attacks will occur in dark areas.

## Results and Discussion

### General patterns of the attacks

**Figure 1:**
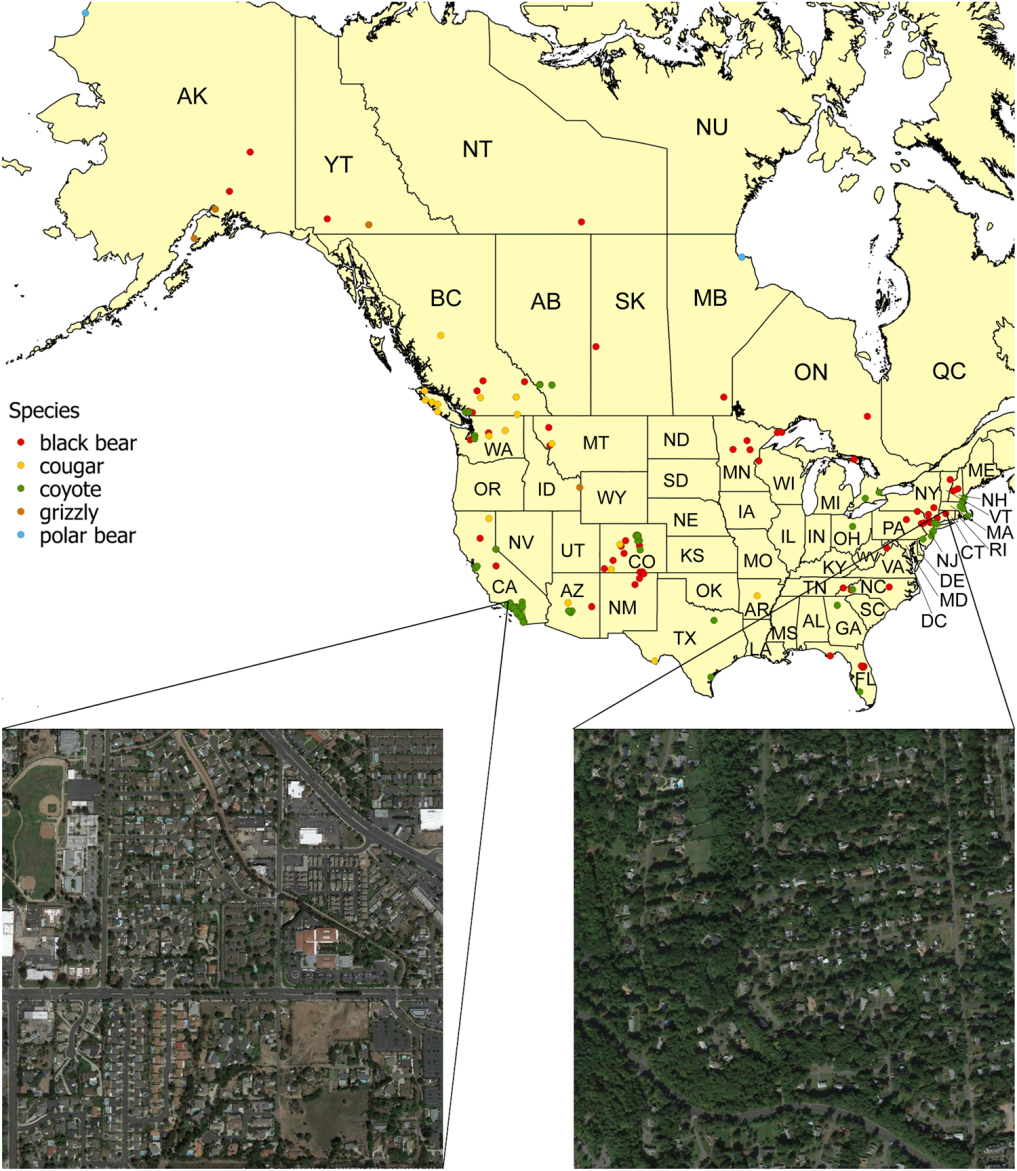
Map of the spatial distribution of wild carnivore attacks on humans recorded in North American urban areas between 1980 and 2016. As an example of the landscape structure analyzed in this study, two 1 km^2^ maps centered at the point of the attack are also shown. The map was created using QGIS software^50^. Boundary layers for Canada and U. S. were obtained from the Statistics Canada Catalogue^51^ and United States Census Bureau^52^ respectively. The two satellite images were obtained from the Google Earth Pro application^53^. Left image: Google Earth 7.3.1. (Imagery Date: April 24, 2014). California, U.S. 33°53′16.00″N, 117°48′40.04″W. Landsat/Copernicus. https://www.google.com/earth/ [September 20, 2017]. Right image: Google Earth 7.3.1. (Imagery Date: September 20, 2013). Connecticut, U.S. 41°47′24.87″N, 72°45′32.29″W. Landsat/Copernicus. https://www.google.com/earth/ [September 20, 2017].

Most of the attacks occurred in California (n = 66, 37%), followed by Colorado (n = 16, 9%), British Columbia (n = 13, 7%) and the other jurisdictions (47%) (Fig. 1 and Supplemental Fig. S1a). The number of attacks recorded in urban areas has increased over time, with similar trends for the different species (Supplemental Fig. S2a). Spring and summer were the seasons showing the highest rates of attack (Supplemental Fig. S2b). Coyotes attacked uniformly throughout the year, with a slight peak during the spring, bear attacks were rare during winter and cougars attacked more often during spring and summer. This seasonal pattern conforms to the species’ biology and confirms what was previously shown in other studies^17,19–21^. Indeed: (a) bears are generally hibernating during winter; and (b) coyotes are rearing their pups during spring, when we observed a slight increase in attacks, and thus they might be in search of additional food and defending their dens during this period^1^, which makes them more likely to be involved in aggressive encounters with humans and pets^22^.

Most of the attacks occurred during the day, especially those involving coyotes and cougars (Supplemental Fig. S3a). This outcome is likely the result of the daily activity of humans in urban areas. Moreover, although coyotes in urban environments have been shown to change their activity patterns to crepuscular and nocturnal to avoid humans^15^, in many cities they have become habituated to people and, consequently, they might have lost their avoidance behavior and returned to being active during the day^15^. On the other hand, black bears tend to be mostly active at night to avoid humans^23,24^.

In general, children (<13 y. o.) were attacked less often than adults (Supplemental Fig. S2c), with a trend towards younger individuals (n = 34 attacks between 0 and 3 years old, n = 16 between 4 and 7 years old and n = 14 between 8 and 11 years old; no attacks were recorded on 9-, 10- and 12-year-old children). Coyotes and cougars attacked children and adults almost equally, while bears attacked considerably more adults than children (Supplemental Fig. S2c). This difference is probably related to the reasons triggering an attack. Indeed, brown and black bears were mainly involved in attacks related to dog presence and anthropogenic food (related to food and trash handling), two scenarios that primarily involve adults, whereas most of the predatory attacks in which victims were prevalently children^25^ were carried out by cougars and coyotes. Additionally, bears attacked more frequently at night, when children are less likely to be found outside than adults”. These patterns also reflect differences in the species’ ecology. While bears are omnivores, cougars are strictly carnivore and coyotes, although they are known to forage on other resources as well^26,27^, are also mainly carnivore. Consequently, we can expect cougars and coyotes to be involved in predatory attacks (and, therefore, attack children) more likely than bears.

The presence of dogs at the moment of the attack was the most prevalent scenario, followed by attacks related to anthropogenic food, predatory motivation and other kinds of scenarios (Supplemental Fig. S2d). Cougar and polar bear attacks were all predatory. Victims of predatory attacks were mainly children (84%), and coyotes were responsible for the majority (63%) of these attacks. People involved in attacks related to dog presence were all adults, which represented the majority of the victims of food-related attacks as well. The high incidence of night attacks when the presence of a dog is involved is probably linked to the late walks that dog owners take in urban areas due to their work schedules and locally hot temperatures during the day^28,29^.

There was only a slight difference between the number of male and female victims (Supplemental Fig. S3b). Most of the victims of black bear attacks were alone, while coyotes attacked unaccompanied people and children in a party nearly equally (Supplemental Fig. S3c).

### Landscape structure and artificial light at the site of the attacks

**Figure 2:**
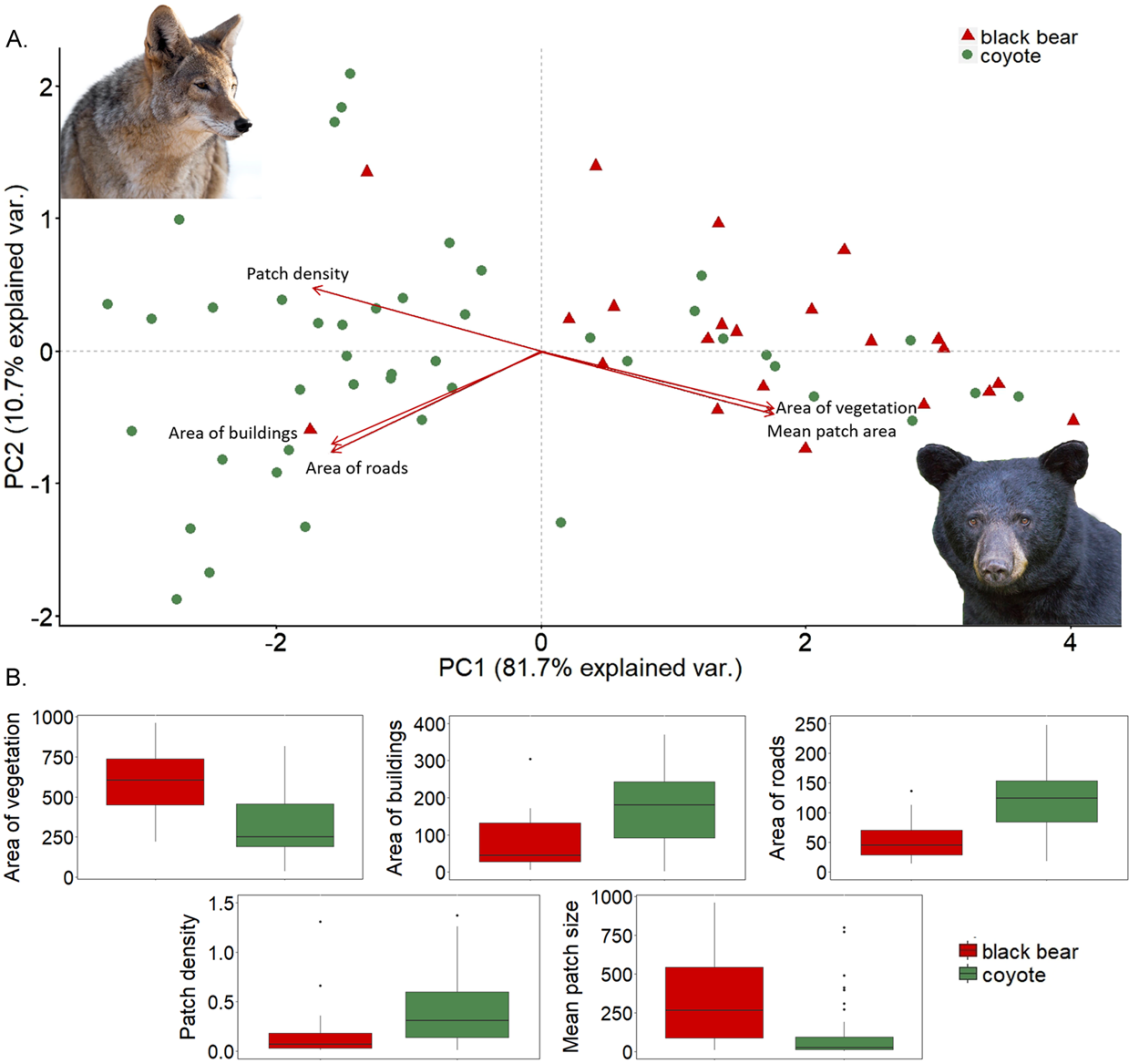
(**A**) Outcome of the PCA run on the 5 landscape variables for black bears (n = 22) and coyotes (n = 47), the species for which the exact location of the attacks was available. Each point represents one attack and arrows show the direction of the variables considered, with variable values increasing according to the direction of the arrow. PC1 and PC2 explained 81.7% and 10.7% of the variance, respectively. (**B**) Boxplots depicting how values of each landscape parameter differ between the two species considered (the coyote photo was downloaded from 123RF ROYALTY FREE STOCK PHOTOS, www.123rf.com, Image ID 52238509, copyright Koji Hirano, https://www.123rf.com/stock-photo/close_up_image_of_coyote.html?oriSearch=a+portrait+showing+the+expressive+eyes+of+a+large+black+bear++ursus+americanus++in+the+mountians&sti=mshqs5mkzdbtehq90h|&mediapopup=52238509; the black bear photo was downloaded from 123RF ROYALTY FREE STOCK PHOTOS, www.123rf.com, Image ID 69859949, copyright cuttsnaturephotography, https://www.123rf.com/stock-photo/a_portrait_showing_the_expressive_eyes_of_a_large_black_bear_(ursus_americanus)_in_the_mountians.html?&sti=mz65utjpox4ztzb2zg|&mediapopup=69859949).

The exact location of the attacks was available for coyotes and black bears only (n_black bear_ = 22, n_coyote_ = 47) and, of these attacks, 15 occurred at night (n_black bear_ = 7, n_coyote_ = 8). Our results were consistent with our initial hypothesis. Indeed, the PCA (Supplemental Tables S1A and S1B) showed a clear difference between attacks by coyotes and black bears in terms of landscape structure (Fig. 2). On one hand, black bear attacks occurred in areas with specific landscape conditions, i.e. (a) few buildings and roads, and (b) dense vegetation cover, which is in line with the ecology of the species both in wildlands and urban areas^11,30,31^, as well as with previous studies which have analyzed the spatial distribution of other types of human-black bear conflicts^2,4,7,32^. These studies suggested that the probability of conflicts with this species was correlated with proximity to large forest patches and intermediate housing densities. This is probably related to the fact that black bears are predominately a forest obligate species^30,32^, although they have been shown to increase selection for human developments during poor food years and the hyperphagia period (summer-fall)^21,31^.

On the other hand, coyotes, a species known to be able to adapt well to urban areas and to tolerate high levels of human disturbance^33^, attacked in a wider range of landscape types than black bears, from areas with high and aggregated cover and few human structures, to extremely urbanized areas with little and fragmented vegetation. Specifically, most of the attacks by this species (~70%) occurred in areas where the vegetation cover was less abundant and more fragmented (i.e., divided into more and smaller patches), and with relatively more buildings and roads, while fewer attacks (~30%) occurred in areas with characteristics similar to those of black bears (i.e., more abundant and aggregated vegetation cover, fewer buildings and roads). Our results are in line with observed patterns related to other types of human-coyote conflicts, which have been shown to be more frequent in developed areas with intermediate housing densities and low vegetation cover than in areas with higher percentages of forest^5,14^. Additionally, Lukasik and Alexander^18^ found that more conflicts occurred where small parks, greenspaces and riparian habitats were present in areas with high human densities.

**Figure 3:**
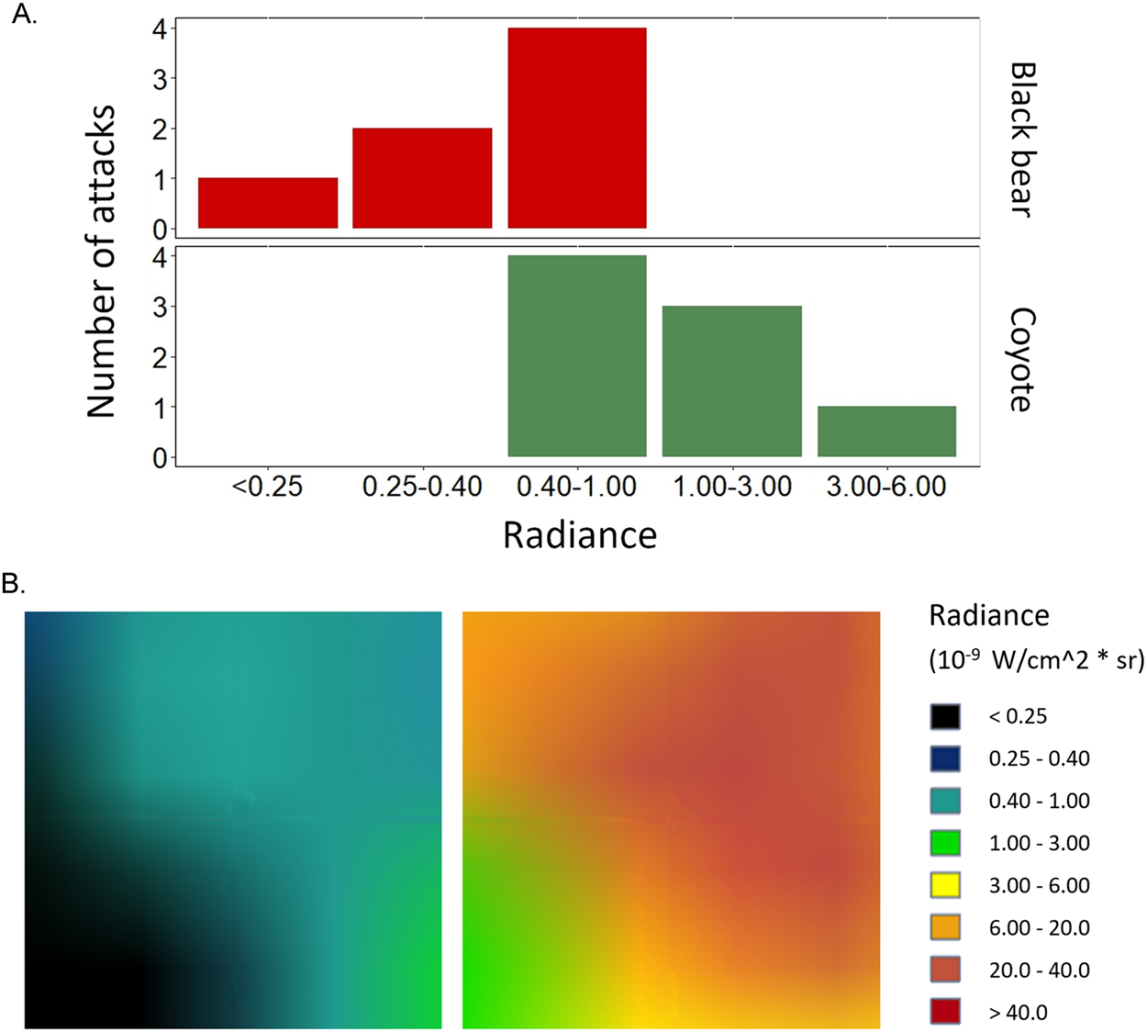
Artificial light conditions for those sites where the attack occurred at night. (**A)** Frequencies of the different ranges of radiance for the two species for which exact locations were available, i.e., coyotes (n = 8) and black bears (n = 7). (**B**) Two 1 km^2^ plots centered at the point of the attack are presented as an example of the artificial light conditions analyzed. Each range of radiance is assigned a different color, from black (radiance <0.25, i.e., no artificial light) to dark-red (radiance >40, i.e., highest amount of artificial light)^46^ (see the main text for more details). The two images were obtained from the website https://www.lightpollutionmap.info.

We found that those attacks that occurred at night took place in areas with a relatively low amount of artificial light (radiance always <6.00 *W/cm*^2^ * sr), with black bear attacks occurring in particularly dark areas (radiance values lower than 1.00 *W/cm*^2^ * sr; Supplemental Table 1C, Fig. 3). This outcome might be related to the recorded abundance of vegetation cover at the locations of black bear attacks. Indeed, we can expect that areas with high vegetation cover are also characterized by lower artificial light than intensely urbanized sectors.

### Are there solutions for this increasing conflict?

#### The role of human behavior in the attacks

Dog presence: It is noteworthy that in at least 20 (66%) of the 33 attacks related to the presence of dogs, humans were not the first target. In these incidents, either the carnivore targeted the dog first and the owner intervened in its defense (most of the cases, 80%, n = 16) or the dog confronted the carnivore, with the owner being subsequently involved in the encounter. Improved public education by local authorities on how to behave with dogs in areas frequented by wild carnivores would certainly help increase public awareness and thus reduce the occurrence of these incidents. As also previously suggested^14,16,17^, keeping dogs on-leash while out walking in areas with carnivores would reduce the number of risky encounters. In the case of coyotes, scaring off the animal with the help of objects has been recommended by wildlife services^16,19,34^. Similarly, keeping dogs inside or in a well-fenced shelter in the yard might help to avoid predatory attempts when the owner is not directly taking care of their dog^16^. Our results suggest that, while for coyotes these precautions should always be taken, i.e. independent of the landscape structure and light conditions, in areas where black bears are present they might be particularly important when the vegetation cover is high and the density of human buildings is low. Additionally, particular attention should be taken at night, especially in areas where artificial illumination is scarce.

Attractants management: The insufficient management of anthropogenic food such as pet food, bird feeders and garbage, both in private properties and public parks, together with the practice of wildlife feeding, are already known to be among the most common causes of human-carnivore conflicts^15,19,20,35^. The proper management of attractants is even more important within urban areas, due to the high number of people potentially exposed to a risky encounter with a wild carnivore. Although significant effort has been made to inform and educate the public on how to reduce attractants, and wildlife feeding has been forbidden in many cities^19,36^, the increasing trend of attacks indicates that current efforts might not be sufficient and more resources should be invested in preventive actions. Additionally, while education and regulations alone might have little effect on changing human behaviour^37,38^, combining these actions with proactive enforcement (e.g. increased patrolling and application of warnings) might prove to be more efficient in altering human behaviour^11,38^.

Predatory attacks on children: Lone children are the preferred target of coyote, cougar and black bear predatory attacks. This kind of attack is the most dangerous and has already been documented in previous studies^15,19,25^. When outside, both in yards and green spaces, children should be continuously supervised by an adult, at a minimum, and never left alone. The presence of an adult may help to reduce the chances of a child being attacked. Additionally, fencing yards and playgrounds in areas where carnivores are present may be an effective precaution to increase child safety.

#### The role of landscape planning

Assuming that both human and carnivore populations will continue to rise in the future, we should expect an increasing overlap between urban areas and carnivore ranges and, therefore, an increase in the number of attacks. The sprawl of human developments towards natural habitats is rapidly rising and residential housing is expected to increase across the landscape, due to homebuyers’ preferences for single-family detached homes^10,39^. Moreover, the recent trend towards “greener” and wildlife-friendly urban landscape design is leading urban planners to promote the inclusion of natural patches and wildlife habitat requirements into the urban matrix^9,40,41^, which may create optimal habitats for some carnivore species. The presence of green spaces and the recent spread of the practice of “wildlife gardening” (i.e., employment of a series of practices aimed at increasing wildlife in gardens) have been shown to provide important benefits to both human health and wildlife biodiversity^6,40,42^. However, practices such as keeping dense vegetation and fruit-trees in yards and green areas, as well as leaving bird feeders outside, are likely to attract wild carnivores and, consequently, may increase the probability of a risky encounter^5,11,32^. These practices should then be avoided in urban areas with resident carnivore populations and/or located near carnivore habitats. Instead, reducing thick vegetation (e.g., dense forests or bushes) to increase visibility and prevent carnivores from using it as shelter, as well as the implementation of fences and improved artificial illumination systems in green areas and yards, can effectively result in increasing both human and pet safety (see also^5,15,34^).

Similarly, in areas scheduled for development, urban planners and homebuyers should be informed of the risk that low-density developments (i.e., sparse housing developments which incorporate large wildland areas) might involve^10^. These kinds of developments, which also include ex-urban and suburban areas, have already been shown to favor the colonization of urban areas by wild carnivores^5,33^ and present a higher concentration of human-wildlife conflicts, especially when situated in proximity to natural areas^4,6,10,14^. In this sense, in terms of land use, our findings support the “land sparing” model, which favors high-density developments in order to preserve wildland^43,44^. This kind of development might be an effective way not only to minimize habitat fragmentation in general, but also to exclude carnivores from urban areas by separating human developments from wildlife habitats and, thus, reduce the occurrence of negative interactions with these species. Finally, we suggest that further studies should investigate whether the attacks are more likely to occur in specific areas within the areas used by the species. This fine-scale analysis would require radiotagging of urban carnivores, which will allow comparing the characteristics of the urban sites where attacks may occur (our results) *vs*. the areas selected by these species.

## Conclusions

Several behavioral and landscape-related factors were recurrent elements in the attacks recorded in North American urban areas. Therefore, effective strategies aimed at benefitting both humans and carnivores will need to combine carnivore knowledge, citizen education and landscape planning. Specifically: (1) because different species attack under different conditions, management plans should be developed according to the species occurring in a given area and generalizations should be avoided; (2) education actions should provide the public with practical information on how to avoid conflicts and how to behave in case of an encounter with a wild carnivore; and (3) landscape planners should work to develop plans able to balance human health, wildlife conservation and conflict risk. Specific landscape modifications and design should thus be employed both in already existing urban green areas and when planning new urban areas.

## Methods

### Collection of records of carnivore attacks on humans in urban areas

We collected reports of wild carnivore attacks on humans resulting in physical injury or death that occurred in urban areas in the United States and Canada from 1980 to 2016. We used attack reports included in the database used in Penteriani *et al*.^17^ by only selecting the attacks which occurred in urban areas within the above-mentioned study area and time period. We included attack reports starting in 1980 because attacks were poorly documented before that year. We then updated the database by adding reports from the years 2014 to 2016.

Our search included the following species: brown bear/grizzly *Ursus arctos*, black bear *Ursus americanus*, polar bear *Ursus maritimus*, cougar *Puma concolor*, grey wolf *Canis lupus* and coyote *Canis latrans*. We attempted to exclude attacks by rabid animals from this work because their behavior is likely atypical. Records of attacks were collected from unpublished reports and PhD/MS theses, webpages, books and scientific articles. To complete the dataset, we also collected news reports from online newspapers. To do this, for each species and area, we searched on an annual basis for news articles on Google using the combination of the following terms: ‘species name’ + ‘attack’, ‘species name’ + ‘attack’ + ‘human’ and ‘species name’ + ‘attack’ + ‘State/province name’ + ‘year’. Because we used several sources, some of the attacks recurred repeatedly during the search, but we used information such as date, location and sex/age of the victims to prevent duplicate records in the dataset. Furthermore, we were able to obtain additional information concerning attacks from the Florida Fish and Wildlife Conservation Commission, Washington Department of Fish and Wildlife, New Mexico Department of Game and Fish and New York Department of Environmental Conservation. This information allowed us to (1) verify if the information we had recorded about the attacks was correct, (2) obtain the exact location of the attacks recorded and (3) obtain new attack reports (if any).

We collected a total of 177 attacks, of which 63% were by coyotes (n = 101), 27% by black bears (n = 44), 7% by cougars *Puma concolor* (n = 12), 2% by polar bears *Ursus maritimus* (n = 4) and 1% by grizzlies *Ursus arctos* (n = 2) (Supplemental Fig. S1b).

For each attack, we recorded the following information: (1) carnivore species; (2) year; (3) month; (4) exact location of the attack; (5) time of the day, which we classified into three categories: twilight, day, night; (6) location of the attack within the urban area: inside home, near home, playground/park, school, others (examples of other locations include: outside a hotel, parking lot, golf course, university campus, on the street); (7) sex and age of the carnivore; (8) sex and age of the victim; (9) party composition, simplified into three categories: (a) victim alone, (b) child –from 0 to 13 years old– in a party of adults, and (c) party of adults, i.e., people >13 years old; (10) end of the attack, i.e., injuries or death; and (11) scenario, i.e., the main factor that could have triggered the attack. We defined four scenarios: (a) predatory, i.e. when the carnivore deliberately attacked and/or killed a human with the presumed purpose of consuming it. Specifically, we considered predatory only those cases where: (1) the human was treated as food (i.e., the person is dragged by the carnivore far from the attack site to a more hidden location such as a forest patch or bushes); (2) the body (of both live and dead victims) is covered with leaves and soil; (3) after its death, the victim is partially consumed; and/or (4) a carnivore has been found near the body^25^; (b) dog-related, i.e., one or more dogs present; (c) anthropogenic food-related, e.g., a carnivore reported feeding on anthropogenic food at the time of the attack or an individual known to be food-conditioned or intentionally fed by humans; and (d) other scenarios, i.e., female with young, aggressive reaction after a sudden encounter, food/territory defense or a wounded animal.

### Characterization of the landscape structure at the site of the attacks

To describe the landscape structure of the attack site, we selected only those attacks for which the exact location was available (39% of the total attacks recorded; an estimated maximum error of ca. 100 m was accepted). The exact locations of the attacks were obtained from the U.S. departmental agencies mentioned above and other sources reporting the precise site of the attack (i.e., providing the address, coordinates or the name of the park/school where the attack took place).

We uploaded the coordinates of each attack into the Google Earth Pro application and selected a plot of 1 km^2^ centered at the point of the attack. We considered 1 km^2^ to be a good trade-off between the accuracy of the location points recorded and the aim of our work, which was to analyze landscape structure in the immediate vicinity of the attack site. Because of the dynamic structure of both natural and human landscapes, for each attack we searched for the map of the year when the attack took place. When the map of the year was not available, we used a map from within 3 years preceding or following the attack. Once the satellite images for each attack location were extracted, we analyzed them by using the image processing software Photoshop CS6 and calculated 5 landscape parameters to both quantify vegetation structure and describe the degree of aggregation of the vegetation in our plots: the area (in m^2^) occupied by (1) vegetation (trees and shrubs), (2) buildings and (3) roads; (4) the vegetation patch density (PD), defined as the number of vegetation patches (i.e. homogeneous areas occupied by vegetation) per unit area, where high values of PD mean a high number of patches per area unit (i.e., highly fragmented vegetation); and (5) the mean patch size in the form of area-weighted mean patch size (AREA_AM), which equals the sum, across all patches, of the patch area multiplied by the proportional abundance of the patch (i.e., patch area divided by the sum of all patch areas). The area-weighted mean patch size (AREA_AM) is less sensitive to small patches than simple mean size and provides a better overall measure of subdivision^45^. PD and AREA_AM were calculated using the area of vegetation and the number of vegetation patches in each plot following Mcgarigal *et al*.^45^. Both metrics were calculated at the class level (i.e., vegetation level), as our landscape area was constant throughout all 1 km^2^ plots.

### Collection of information related to artificial light during night attacks

For those attacks that occurred at night and for which we had the exact location (n_black bear_ = 7, n_coyote_ = 8), we analyzed the amount of artificial light available near the attack site. Specifically, we extracted a map of artificial light at the attack site (as with the landscape parameters, we considered an area of 1 km^2^ centered at the point of the attack) from the website https://www.lightpollutionmap.info/. This website provides a world atlas of artificial night sky radiance (in *W/cm*^2^ * sr, where W = watt and sr = steradian or square radian, i.e. the International System of Units of solid angles that quantifies planar angles, which is used to measure the luminous intensity of a light source)^46^, where different ranges of radiance are represented by different colors. Specifically, low values of radiance correspond to lower amounts of artificial light, with radiance values <0.25 considered as a typical moonless night sky background far from the Milky Way, zodiacal and artificial light (artificial sky brightness <1% of the natural background)^46^. The atlas includes maps from 2010 to 2017 and, as with the landscape metrics, when the map of the year was not available we used a map from within 3 years preceding or following the attack. Once the images were extracted, we calculated the area occupied by each color (i.e. range of radiance) using Photoshop CS6 and calculated the mean radiance of each map.

### Data analysis

#### Landscape structure and artificial light at the site of the attacks

Since the landscape parameters estimated were correlated (Spearman rank correlation r_s_ always >0.70, P < 0.001), after log-transforming PD and AREA_AM, we ran a Principal Component Analysis (PCA) including the 5 variables. We then built a set of competing models which included the number of attacks per species as the response variable and principal component 1 (PC1) and principal component 2 (PC2) obtained from the PCA as explanatory variables. We finally built a second set of models, again with the number of attacks per species as the response variable, but now including radiance (i.e., our proxy of the artificial light conditions) as the explanatory variable. In both sets of models, as our response variable was categorical and had 2 levels (i.e., either attacks by coyotes or attacks by black bears), we built Generalized Linear Models (GLMs) with a binomial distribution. We performed model selection based on the Akaike’s Information Criterion corrected for small sample sizes (AICc; Burnham & Anderson)^47^ and calculated two additional statistics for each model: ΔAICc and AICc weights, which indicate the probability that the model selected was the best among the competing candidates^48^. We considered models with ΔAICc values lower than 2 as competitive. All statistical analyses were performed using R 3.2.5 statistical software^49^.

## Electronic supplementary material

Supplementary Information
