# Supplementary material for: Patterns of wild carnivore attacks on humans in urban areas
Source: Sci Rep. 2018 Dec 7;8:17728. doi: 10.1038/s41598-018-36034-7 (PMC6286335; doi:10.1038/s41598-018-36034-7)
Supplement: Supplementary file 1 — Supplementary Information [file 41598_2018_36034_MOESM1_ESM.pdf]

Supplementary information

Patterns of wild carnivore attacks on humans in urban areas

Giulia Bombieri[1,2], María del Mar Delgado[1], Luca Francesco Russo[1], Pedro José Garrote[3], José Vicente López-Bao[1], José M. Fedriani[3] and Vincenzo Penteriani[1,4]

[1]Research Unit of Biodiversity (UMIB, UO-CSIC-PA), Oviedo University - Campus Mieres, Mieres, Spain

[2]Museo delle Scienze, Sezione Zoologia dei Vertebrati, Corso del Lavoro e della Scienza 3, I-38123 Trento, Italy

[3]Centre for Applied Ecology "Prof. Baeta Neves"/InBIO, Institute of Agronomy, University of Lisbon, Tapada da Ajuda, Lisboa, Portugal

[4] Instituto Pirenaico de Ecología, C.S.I.C., Avda. Nuestra Señora de la Victoria 16, 22700 Jaca, Spain

Supplemental Figure S1. a. Urban areas of the U.S. and Canada where large carnivore attacks occurred between 1980 and 2016; **b**. Species of large carnivores involved in attacks on humans in urban areas of the U.S. and Canada between 1980 and 2016. The conflict end (i.e., injury or death) is also shown.

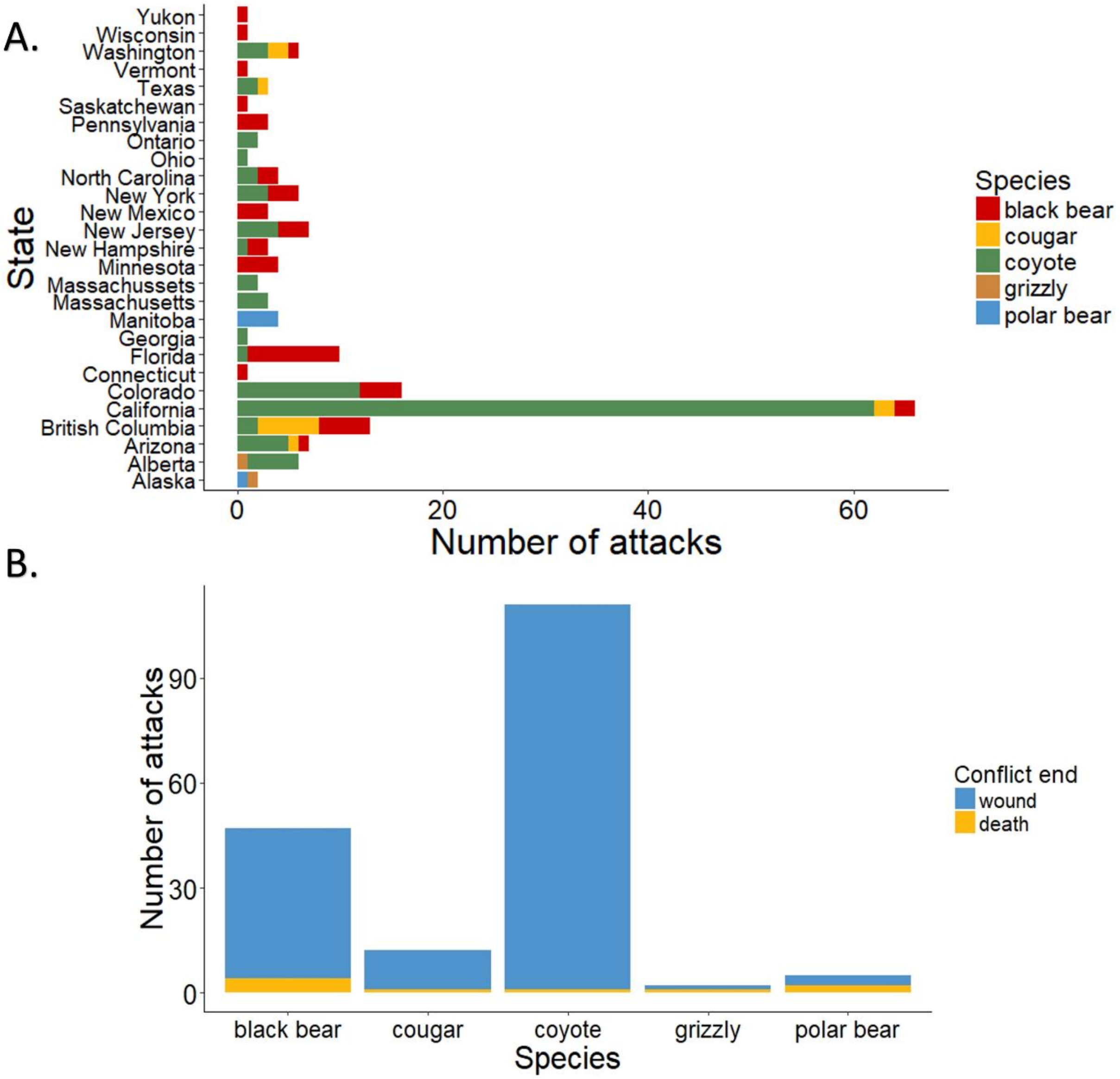

Supplemental Figure S2. Features of large carnivore attacks on humans in North American urban areas from 1980 to 2016. **a**. Temporal trends, showing an increase in recent times; **b**. Seasonal patterns; **c.** Age of the victims; and **d**. Scenario of the attacks, i.e., factors triggering the attacks.

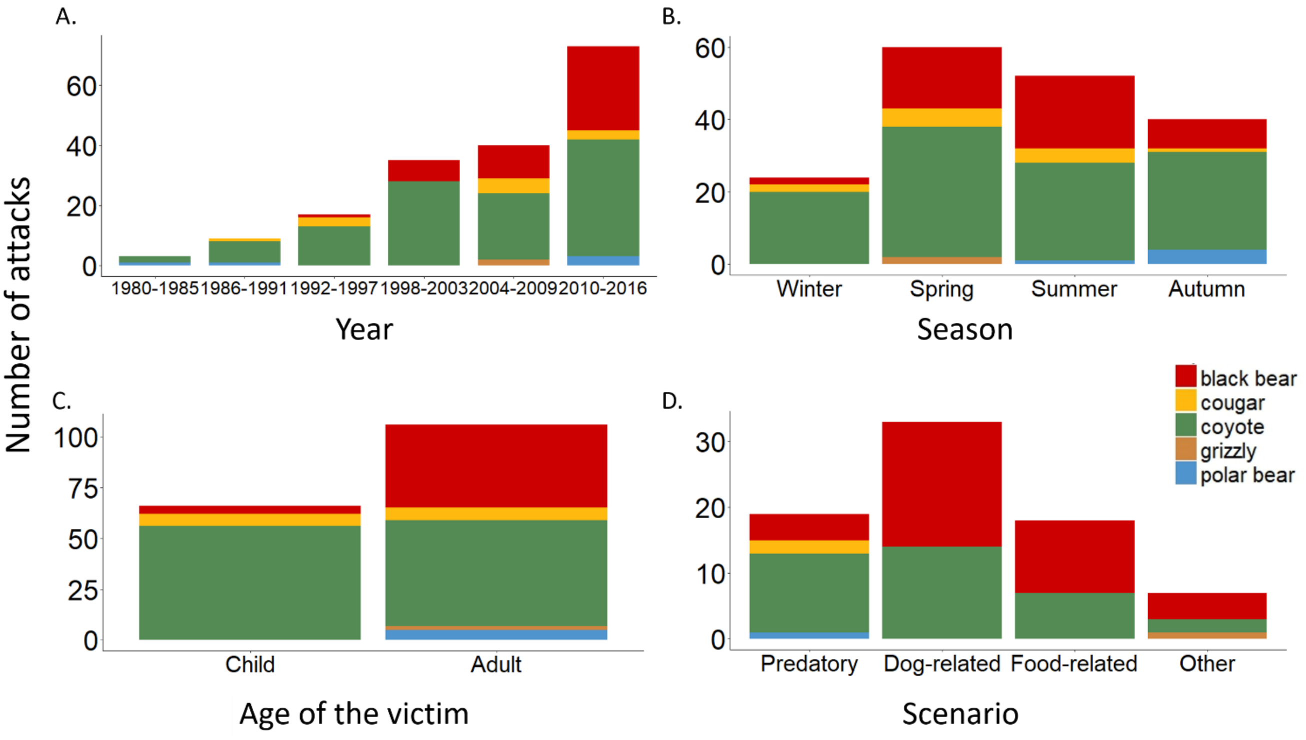

Supplemental Figure S3. a. Dial patterns of large carnivore attacks on humans recorded in North American urban areas between 1980 and 2016; **b**. Sex of the victims; **c**. Composition of parties involved in the attacks.

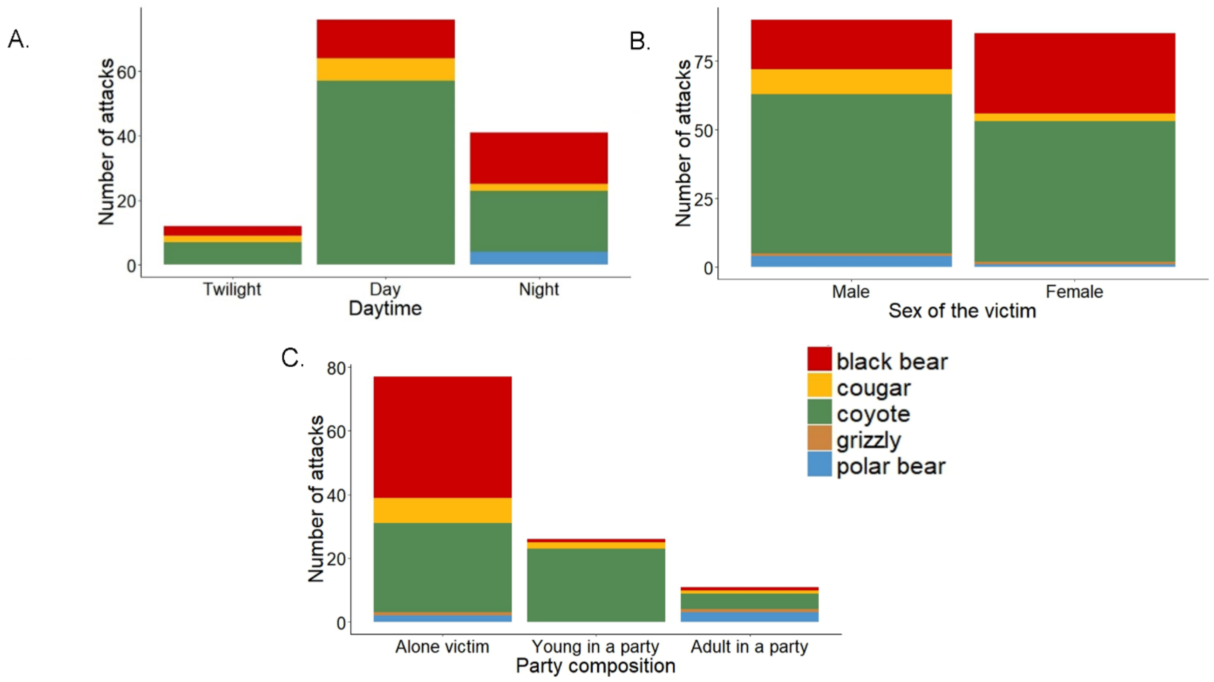

Supplementary Table S1. A. Comparison of the competing models built to analyze the landscape structure at the site of the attacks based on values of AICc, ΔAICc and AICc weights (n = 69). Competitive models are ranked from the lowest (best model) to the highest AICc value. Summary of fitted parameters is shown for models with ΔAICc < 2.

Response variable: number of attacks per large carnivore species (2 levels: attacks by coyotes and attacks by black bears) – binomial distribution error. Deviance = 0.288.

COMPETING MODELS		***β***	***SE***	***p***	***AICc***	***ΔAICc***	***AICc weights***
PC1					*67.84*	*0.00*	*0.50*
	Intercept	*1.079*	*0.343*	*0.00164*			
	PC1	*-0.722*	*0.182*	*7.08e-05*			
PC1+PC2					*67.84*	*0.00*	*0.50*
	Intercept	*1.228*	*0.391*	*0.001672*			
	PC1	*-0.798*	*0.205*	*0.000101*			
	PC2	*-0.762*	*0.527*	*0.148228*			
NULL					*88.45*	*20.60*	*0.00*
PC2					*89.91*	*22.07*	*0.00*

B. Summary of the output of the PCA run on the landscape variables. Importance of each component and loadings are reported.

**IMPORTANCE OF COMPONENTS**	**PC1**	**PC2**	**PC3**	**PC4**	**PC5**
Standard deviation	2.02	0.73	0.46	0.32	0.26
Proportion of Variance	0.82	0.11	0.04	0.02	0.01
Cumulative Proportion	0.82	0.93	0.97	0.99	1.00
**LOADINGS**					
Area of vegetation	0.466	-0.328	-0.196	0.427	0.675
Area of buildings	-0.422	-0.573	-0.657	-0.247	
Area of roads	-0.424	-0.560	0.659	0.265	
Mean patch size	0.465	-0.356		0.335	-0.732
Patch density	-0.458	0.351	-0.295	0.758	

C. Comparison of the competing models built to analyze conditions of artificial light at the site of the attacks based on values of AICc, ΔAICc and AICc weights (n = 15). Competitive models are ranked from the lowest (best model) to the highest AICc value. Summary of fitted parameters is shown for models with ΔAICc < 2.

Response variable: number of attacks per large carnivore species (2 levels: attacks by coyotes and attacks by black bears) – binomial distribution error. Deviance = 0.473.

COMPETING MODELS		***β***	***SE***	***p***	***AICc***	***ΔAICc***	***AICc weights***
RADIANCE	*Intercept*	*-4.372*	*2.754*	*0.1124*	*15.92*	*0.00*	*0.97*
	Radiance	*0.328*	*0.197*	*0.0951*			
NULL					*23.04*	*7.11*	*0.03*
